# (μ-4-Methyl­benzene­thiol­ato-κ^2^ *S*:*S*)bis­[carbon­yl(η^5^-cyclo­penta­dien­yl)molybdenum(II)]

**DOI:** 10.1107/S1600536808010751

**Published:** 2008-04-23

**Authors:** Richard Chee Seng Wong, Mei Lee Ooi, Seik Weng Ng

**Affiliations:** a Department of Chemistry, University of Malaya, 50603 Kuala Lumpur, Malaysia

## Abstract

The asymmetric unit of the title compound, [Mo_2_(C_5_H_5_)_2_(C_7_H_7_S)_2_(CO)_2_], consists of two half-mol­ecules, each molecule lying on a centre of symmetry. The thiol­ate groups function as bridges between the Mo^II^ atoms, which adopt a quasi-octa­hedral geometry. In the octa­hedral environment the two ligating S atoms are in a *cis* arrangement.

## Related literature

For related literature on unsubstituted [MoCp(CO)(μ-SC_6_H_5_)]_2_, see: Song *et al.* (1997 ▶). The 4-methyl-substituted compound was characterized spectroscopically, see: Benson *et al.* (1980 ▶).
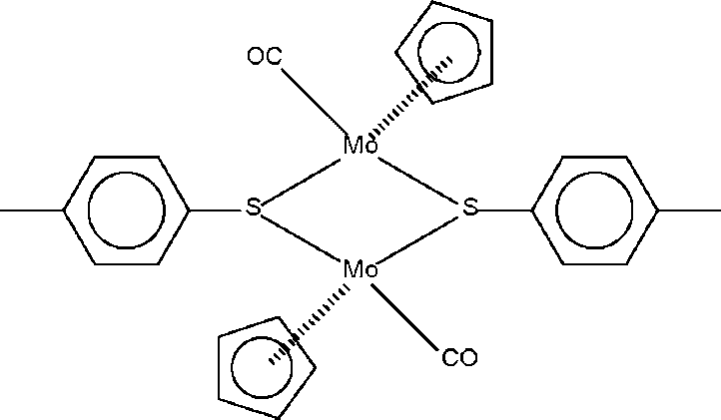


## Experimental

### 

#### Crystal data

[Mo_2_(C_5_H_5_)_2_(C_7_H_7_S)_2_(CO)_2_]
*M* *_r_* = 624.45
Monoclinic, 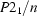

*a* = 13.245 (2) Å
*b* = 10.135 (1) Å
*c* = 18.042 (2) Å
β = 104.901 (2)°
*V* = 2340.4 (5) Å^3^
*Z* = 4
Mo *K*α radiation
μ = 1.27 mm^−1^
*T* = 100 (2) K
0.06 × 0.06 × 0.06 mm

#### Data collection

Bruker APEXII diffractometer
Absorption correction: multi-scan (*SADABS*; Sheldrick, 1996 ▶) *T*_min_ = 0.758, *T*_max_ = 0.928
14384 measured reflections
5374 independent reflections
3915 reflections with *I* > 2σ(*I*)
*R*_int_ = 0.048

#### Refinement

*R*[*F* ^2^ > 2σ(*F* ^2^)] = 0.043
*wR*(*F* ^2^) = 0.104
*S* = 1.03
5374 reflections
291 parameters
H-atom parameters constrained
Δρ_max_ = 2.60 e Å^−3^
Δρ_min_ = −1.77 e Å^−3^

### 

Data collection: *APEX2* (Bruker, 2007 ▶); cell refinement: *SAINT* (Bruker, 2007 ▶); data reduction: *SAINT*; program(s) used to solve structure: *SHELXS97* (Sheldrick, 2008 ▶); program(s) used to refine structure: *SHELXL97* (Sheldrick, 2008 ▶); molecular graphics: *X-SEED* (Barbour, 2001 ▶); software used to prepare material for publication: *publCIF* (Westrip, 2008 ▶).

## Supplementary Material

Crystal structure: contains datablocks global, I. DOI: 10.1107/S1600536808010751/ci2579sup1.cif

Structure factors: contains datablocks I. DOI: 10.1107/S1600536808010751/ci2579Isup2.hkl

Additional supplementary materials:  crystallographic information; 3D view; checkCIF report

## Figures and Tables

**Table 1:** Selected bond lengths (Å)

Mo1—S1	2.424 (1)
Mo1—S1^i^	2.425 (1)
Mo2—S2	2.430 (1)
Mo2—S2^ii^	2.419 (1)

Symmetry codes: (i) 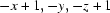; (ii) 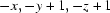.

**Fig. 1.**
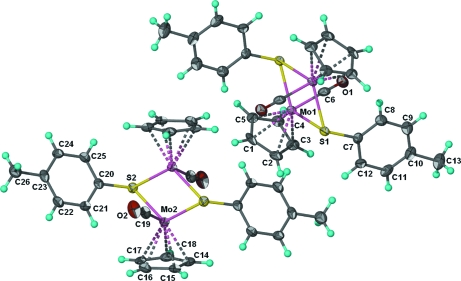
Displacement ellipsoid plot (Barbour, 2001) of the two independent dimeric [Mo(C5H5)(CO)(C7H7S)]2 molecules, drawn at the 70% probability level. H atoms are drawn as spheres of arbitrary radii. For molecules with Mo1 and Mo2, the unlabeled atoms are related to the labeled ones by (1 - x, -y, 1 - z) and (-x, 1 - y, 1 - z), respectively.

## supplementary crystallographic information

### Comment

The title compound was characterized spectroscopically in a study on [MoCp(CO)(µ-SAr]_2_ type of compounds. The compound was synthesized by treating MoClCp(CO)_3_ with 4-tolyl mercaptan (Benson *et al.*, 1980). We have obtained the title compound (Fig. 1) as the unexpected product in the reaction of [(η5-C_5_H_5_)Mo(CO)_2_]_2_ with deuterated *o*-P(PhSCD_3_)_3_. Under the conditions of the reaction, the *ortho*-SD_3_ groups of the phosphine have been rearranged to the *para* position. The rearrangement is then followed by an exchange of the deuterium atoms by hydrogen atoms; the absence of deuterium in the compound is confirmed by ^1^H NMR spectroscopic analysis.

### Experimental

A reddish-brown suspension of [(η5-C_5_H_5_)Mo(CO)_2_]_2_ (100 mg, 0.23 mmol) and an equivalent quantity of the deuterated phosphine, *o*-P(PhSCD_3_)_3_ (94 mg, 0.23 mol), was heated at 383 K for 14.5 h under argon. The mixture was filtered and then absorbed onto Celite (1.5 g). The slurry was evacuated to dryness and loaded onto a silica gel column (9.5 cm *x* 2.0 cm) in *n*-hexane. The title compound was eluted with *n*-hexane-toluene (1:4, 50 ml) as a greenish-brown fraction (60.0 mg, 0.096 mmol, 40% yield). The dichroic compound was recrystallized from an *n*-hexane-toluene mixture and crystals were isolated after 2 d.

### Refinement

H-atoms were placed in calculated positions (C—H = 0.95–1.00 Å) and were included in the refinement in the riding-model approximation, with *U_iso_*(H) = 1.2–1.5*U_eq_*(C). The final difference Fourier map had a peak at 0.80 Å and a deep hole at 0.68 Å from Mo2.

### Crystal data

**Table:**

[Mo_2_(C_5_H_5_)_2_(C_7_H_7_S)_2_(CO)_2_]	*F*_000_ = 1248
*M**_r_* = 624.45	*D*_x_ = 1.772 Mg m^−^^3^
Monoclinic, *P*2_1_/*n*	Mo *K*α radiation λ = 0.71073 Å
Hall symbol: -P 2yn	Cell parameters from 2210 reflections
*a* = 13.245 (2) Å	θ = 2.3–24.8º
*b* = 10.135 (1) Å	µ = 1.27 mm^−^^1^
*c* = 18.042 (2) Å	*T* = 100 (2) K
β = 104.901 (2)º	Cube, green–brown
*V* = 2340.4 (5) Å^3^	0.06 × 0.06 × 0.06 mm
*Z* = 4	

### Data collection

**Table:**

Bruker APEXII diffractometer	5374 independent reflections
Radiation source: fine-focus sealed tube	3915 reflections with *I* > 2σ(*I*)
Monochromator: graphite	*R*_int_ = 0.048
*T* = 100(2) K	θ_max_ = 27.5º
φ and ω scans	θ_min_ = 1.7º
Absorption correction: multi-scan(SADABS; Sheldrick, 1996)	*h* = −17→16
*T*_min_ = 0.758, *T*_max_ = 0.928	*k* = −7→13
14384 measured reflections	*l* = −23→23

### Refinement

**Table:**

Refinement on *F*^2^	Secondary atom site location: difference Fourier map
Least-squares matrix: full	Hydrogen site location: inferred from neighbouring sites
*R*[*F*^2^ > 2σ(*F*^2^)] = 0.043	H-atom parameters constrained
*wR*(*F*^2^) = 0.104	*w* = 1/[σ^2^(*F*_o_^2^) + (0.0453*P*)^2^ + 1.5589*P*] where *P* = (*F*_o_^2^ + 2*F*_c_^2^)/3
*S* = 1.03	(Δ/σ)_max_ = 0.001
5374 reflections	Δρ_max_ = 2.60 e Å^−^^3^
291 parameters	Δρ_min_ = −1.77 e Å^−^^3^
Primary atom site location: structure-invariant direct methods	Extinction correction: none

### Fractional atomic coordinates and isotropic or equivalent isotropic displacement parameters (Å^2^)

**Table:**

	*x*	*y*	*z*	*U*_iso_*/*U*_eq_	
Mo1	0.53951 (3)	0.10457 (4)	0.53870 (2)	0.01595 (11)	
Mo2	−0.01435 (3)	0.62084 (4)	0.47776 (2)	0.02086 (11)	
S1	0.49151 (8)	0.09871 (11)	0.39974 (6)	0.0174 (2)	
S2	0.00847 (9)	0.55650 (11)	0.61079 (6)	0.0195 (2)	
O1	0.7457 (2)	−0.0389 (3)	0.53895 (18)	0.0277 (8)	
O2	−0.2474 (2)	0.5238 (4)	0.4399 (2)	0.0345 (9)	
C1	0.4768 (4)	0.2943 (4)	0.5899 (3)	0.0261 (11)	
H1	0.4026	0.3118	0.5901	0.031*	
C2	0.5256 (4)	0.3337 (4)	0.5324 (3)	0.0262 (11)	
H2	0.4922	0.3862	0.4856	0.031*	
C3	0.6331 (4)	0.2982 (4)	0.5566 (3)	0.0235 (10)	
H3	0.6886	0.3237	0.5309	0.028*	
C4	0.6493 (3)	0.2359 (4)	0.6296 (2)	0.0209 (10)	
H4	0.7183	0.2086	0.6636	0.025*	
C5	0.5531 (4)	0.2342 (4)	0.6494 (2)	0.0223 (10)	
H5	0.5420	0.2022	0.6991	0.027*	
C6	0.6654 (4)	0.0091 (4)	0.5356 (2)	0.0212 (10)	
C7	0.6066 (3)	0.1070 (5)	0.3651 (2)	0.0188 (9)	
C8	0.6512 (4)	−0.0029 (5)	0.3415 (3)	0.0253 (10)	
H8	0.6245	−0.0882	0.3471	0.030*	
C9	0.7354 (4)	0.0111 (5)	0.3093 (3)	0.0261 (11)	
H9	0.7648	−0.0653	0.2926	0.031*	
C10	0.7776 (4)	0.1338 (5)	0.3010 (3)	0.0247 (10)	
C11	0.7337 (4)	0.2431 (5)	0.3263 (3)	0.0272 (11)	
H11	0.7625	0.3279	0.3226	0.033*	
C12	0.6477 (3)	0.2307 (5)	0.3573 (2)	0.0213 (10)	
H12	0.6173	0.3072	0.3730	0.026*	
C13	0.8678 (4)	0.1468 (6)	0.2643 (3)	0.0355 (13)	
H13A	0.9059	0.2287	0.2817	0.053*	
H13B	0.8410	0.1486	0.2084	0.053*	
H13C	0.9150	0.0714	0.2792	0.053*	
C14	0.0611 (4)	0.7759 (5)	0.4160 (3)	0.0269 (11)	
H14	0.1135	0.7584	0.3860	0.032*	
C15	−0.0495 (4)	0.7844 (5)	0.3857 (3)	0.0258 (11)	
H15	−0.0881	0.7726	0.3307	0.031*	
C16	−0.0927 (4)	0.8291 (5)	0.4449 (3)	0.0279 (11)	
H16	−0.1679	0.8510	0.4395	0.033*	
C17	−0.0100 (4)	0.8469 (4)	0.5119 (3)	0.0302 (12)	
H17	−0.0172	0.8827	0.5619	0.036*	
C18	0.0835 (4)	0.8158 (5)	0.4944 (3)	0.0276 (11)	
H18	0.1549	0.8274	0.5295	0.033*	
C19	−0.1602 (4)	0.5542 (5)	0.4557 (3)	0.0279 (11)	
C20	−0.1100 (3)	0.5593 (4)	0.6420 (2)	0.0178 (9)	
C21	−0.1885 (3)	0.6503 (4)	0.6146 (3)	0.0225 (10)	
H21	−0.1827	0.7113	0.5759	0.027*	
C22	−0.2757 (4)	0.6521 (5)	0.6437 (3)	0.0232 (10)	
H22	−0.3293	0.7148	0.6242	0.028*	
C23	−0.2869 (3)	0.5659 (5)	0.6999 (3)	0.0217 (10)	
C24	−0.2062 (3)	0.4753 (4)	0.7287 (2)	0.0206 (10)	
H24	−0.2118	0.4152	0.7678	0.025*	
C25	−0.1182 (3)	0.4732 (4)	0.7002 (2)	0.0199 (9)	
H25	−0.0633	0.4126	0.7206	0.024*	
C26	−0.3837 (4)	0.5654 (5)	0.7300 (3)	0.0282 (11)	
H26A	−0.4087	0.6561	0.7318	0.042*	
H26B	−0.3666	0.5276	0.7818	0.042*	
H26C	−0.4383	0.5124	0.6961	0.042*	

### Atomic displacement parameters (Å^2^)

**Table:**

	*U*^11^	*U*^22^	*U*^33^	*U*^12^	*U*^13^	*U*^23^
Mo1	0.0185 (2)	0.01103 (19)	0.01885 (19)	−0.00035 (15)	0.00568 (15)	−0.00125 (15)
Mo2	0.0320 (2)	0.01180 (19)	0.0223 (2)	0.00178 (17)	0.01341 (17)	0.00156 (16)
S1	0.0177 (5)	0.0141 (5)	0.0211 (5)	0.0004 (4)	0.0062 (4)	−0.0003 (4)
S2	0.0217 (6)	0.0178 (6)	0.0203 (5)	−0.0011 (4)	0.0077 (4)	−0.0015 (4)
O1	0.0218 (18)	0.0253 (19)	0.039 (2)	0.0060 (15)	0.0126 (15)	0.0047 (15)
O2	0.0170 (18)	0.042 (2)	0.043 (2)	−0.0054 (16)	0.0061 (15)	−0.0146 (18)
C1	0.031 (3)	0.014 (2)	0.035 (3)	0.004 (2)	0.011 (2)	−0.011 (2)
C2	0.042 (3)	0.011 (2)	0.025 (2)	0.000 (2)	0.007 (2)	−0.0026 (19)
C3	0.031 (3)	0.015 (2)	0.023 (2)	−0.008 (2)	0.005 (2)	−0.0039 (18)
C4	0.023 (2)	0.016 (2)	0.021 (2)	−0.0067 (19)	0.0008 (18)	−0.0057 (18)
C5	0.033 (3)	0.014 (2)	0.021 (2)	−0.0059 (19)	0.009 (2)	−0.0046 (18)
C6	0.030 (3)	0.013 (2)	0.020 (2)	−0.005 (2)	0.006 (2)	0.0012 (18)
C7	0.017 (2)	0.022 (2)	0.018 (2)	0.0009 (19)	0.0057 (17)	0.0018 (19)
C8	0.027 (3)	0.019 (2)	0.032 (3)	−0.001 (2)	0.012 (2)	0.001 (2)
C9	0.024 (3)	0.028 (3)	0.028 (3)	−0.001 (2)	0.009 (2)	−0.003 (2)
C10	0.025 (2)	0.030 (3)	0.020 (2)	−0.008 (2)	0.0079 (19)	−0.003 (2)
C11	0.033 (3)	0.027 (3)	0.022 (2)	−0.010 (2)	0.010 (2)	0.001 (2)
C12	0.029 (2)	0.017 (2)	0.018 (2)	0.0010 (19)	0.0062 (19)	0.0025 (18)
C13	0.037 (3)	0.040 (3)	0.035 (3)	−0.011 (2)	0.019 (2)	−0.009 (2)
C14	0.035 (3)	0.017 (2)	0.032 (3)	0.000 (2)	0.016 (2)	0.008 (2)
C15	0.038 (3)	0.015 (2)	0.028 (3)	0.005 (2)	0.013 (2)	0.0070 (19)
C16	0.030 (3)	0.016 (2)	0.042 (3)	0.004 (2)	0.017 (2)	0.005 (2)
C17	0.062 (4)	0.008 (2)	0.030 (3)	−0.002 (2)	0.027 (3)	0.0018 (19)
C18	0.039 (3)	0.015 (2)	0.030 (3)	−0.006 (2)	0.010 (2)	0.004 (2)
C19	0.036 (3)	0.023 (3)	0.028 (3)	0.003 (2)	0.015 (2)	−0.003 (2)
C20	0.019 (2)	0.015 (2)	0.019 (2)	−0.0018 (17)	0.0052 (18)	−0.0048 (17)
C21	0.030 (3)	0.017 (2)	0.023 (2)	0.0026 (19)	0.011 (2)	0.0028 (18)
C22	0.028 (2)	0.018 (2)	0.024 (2)	0.005 (2)	0.0067 (19)	−0.0032 (19)
C23	0.024 (2)	0.019 (2)	0.023 (2)	−0.0015 (19)	0.0082 (19)	−0.0089 (19)
C24	0.028 (2)	0.014 (2)	0.021 (2)	−0.0013 (19)	0.0070 (19)	0.0002 (18)
C25	0.022 (2)	0.016 (2)	0.021 (2)	0.0028 (18)	0.0041 (18)	−0.0015 (18)
C26	0.025 (3)	0.026 (3)	0.035 (3)	0.000 (2)	0.010 (2)	−0.002 (2)

### Geometric parameters (Å, °)

**Table:**

Mo1—C6	1.941 (5)	C8—C9	1.390 (6)
Mo1—C3	2.299 (4)	C8—H8	0.95
Mo1—C4	2.312 (4)	C9—C10	1.388 (7)
Mo1—C2	2.330 (5)	C9—H9	0.95
Mo1—C5	2.359 (4)	C10—C11	1.381 (7)
Mo1—C1	2.374 (4)	C10—C13	1.513 (6)
Mo1—S1	2.424 (1)	C11—C12	1.398 (6)
Mo1—S1^i^	2.425 (1)	C11—H11	0.95
Mo1—Mo1^i^	2.6052 (8)	C12—H12	0.95
Mo2—C19	1.988 (5)	C13—H13A	0.98
Mo2—C14	2.298 (5)	C13—H13B	0.98
Mo2—C15	2.308 (4)	C13—H13C	0.98
Mo2—C18	2.340 (5)	C14—C15	1.428 (7)
Mo2—C16	2.359 (5)	C14—C18	1.428 (7)
Mo2—C17	2.370 (5)	C14—H14	1.00
Mo2—S2	2.430 (1)	C15—C16	1.410 (6)
Mo2—S2^ii^	2.419 (1)	C15—H15	1.00
Mo2—Mo2^ii^	2.5751 (8)	C16—C17	1.419 (7)
S1—C7	1.792 (4)	C16—H16	1.00
S1—Mo1^i^	2.4252 (12)	C17—C18	1.391 (7)
S2—C20	1.799 (4)	C17—H17	1.00
S2—Mo2^ii^	2.4192 (12)	C18—H18	1.00
O1—C6	1.157 (5)	C20—C21	1.382 (6)
O2—C19	1.159 (6)	C20—C25	1.391 (6)
C1—C5	1.409 (6)	C21—C22	1.387 (6)
C1—C2	1.414 (7)	C21—H21	0.95
C1—H1	1.00	C22—C23	1.376 (6)
C2—C3	1.423 (6)	C22—H22	0.95
C2—H2	1.00	C23—C24	1.403 (6)
C3—C4	1.426 (6)	C23—C26	1.516 (6)
C3—H3	1.00	C24—C25	1.390 (6)
C4—C5	1.410 (6)	C24—H24	0.95
C4—H4	1.00	C25—H25	0.95
C5—H5	1.00	C26—H26A	0.98
C7—C8	1.380 (6)	C26—H26B	0.98
C7—C12	1.388 (6)	C26—H26C	0.98
			
C6—Mo1—C3	89.65 (18)	C4—C3—H3	126.1
C6—Mo1—C4	85.36 (17)	Mo1—C3—H3	126.1
C3—Mo1—C4	36.02 (15)	C5—C4—C3	107.9 (4)
C6—Mo1—C2	123.59 (18)	C5—C4—Mo1	74.2 (2)
C3—Mo1—C2	35.81 (16)	C3—C4—Mo1	71.5 (2)
C4—Mo1—C2	59.25 (16)	C5—C4—H4	125.8
C6—Mo1—C5	115.04 (17)	C3—C4—H4	125.8
C3—Mo1—C5	58.96 (16)	Mo1—C4—H4	125.8
C4—Mo1—C5	35.11 (15)	C1—C5—C4	108.6 (4)
C2—Mo1—C5	58.27 (16)	C1—C5—Mo1	73.3 (2)
C6—Mo1—C1	143.52 (17)	C4—C5—Mo1	70.6 (2)
C3—Mo1—C1	58.91 (17)	C1—C5—H5	125.6
C4—Mo1—C1	58.47 (16)	C4—C5—H5	125.6
C2—Mo1—C1	34.99 (16)	Mo1—C5—H5	125.6
C5—Mo1—C1	34.63 (16)	O1—C6—Mo1	173.3 (4)
C6—Mo1—S1	87.53 (13)	C8—C7—C12	119.1 (4)
C3—Mo1—S1	98.92 (11)	C8—C7—S1	122.7 (4)
C4—Mo1—S1	134.23 (11)	C12—C7—S1	118.0 (3)
C2—Mo1—S1	88.70 (12)	C7—C8—C9	120.0 (4)
C5—Mo1—S1	146.32 (12)	C7—C8—H8	120.0
C1—Mo1—S1	113.43 (12)	C9—C8—H8	120.0
C6—Mo1—S1^i^	80.65 (13)	C10—C9—C8	121.7 (5)
C3—Mo1—S1^i^	144.04 (11)	C10—C9—H9	119.2
C4—Mo1—S1^i^	108.29 (11)	C8—C9—H9	119.2
C2—Mo1—S1^i^	148.06 (12)	C11—C10—C9	117.9 (4)
C5—Mo1—S1^i^	94.04 (11)	C11—C10—C13	121.4 (4)
C1—Mo1—S1^i^	113.10 (12)	C9—C10—C13	120.7 (4)
S1—Mo1—S1^i^	115.00 (3)	C10—C11—C12	121.0 (5)
C6—Mo1—Mo1^i^	78.96 (13)	C10—C11—H11	119.5
C3—Mo1—Mo1^i^	153.86 (12)	C12—C11—H11	119.5
C4—Mo1—Mo1^i^	160.22 (12)	C7—C12—C11	120.2 (4)
C2—Mo1—Mo1^i^	140.25 (12)	C7—C12—H12	119.9
C5—Mo1—Mo1^i^	147.12 (11)	C11—C12—H12	119.9
C1—Mo1—Mo1^i^	137.32 (12)	C10—C13—H13A	109.5
S1—Mo1—Mo1^i^	57.53 (3)	C10—C13—H13B	109.5
S1^i^—Mo1—Mo1^i^	57.47 (3)	H13A—C13—H13B	109.5
C19—Mo2—C14	131.04 (19)	C10—C13—H13C	109.5
C19—Mo2—C15	95.41 (19)	H13A—C13—H13C	109.5
C14—Mo2—C15	36.11 (16)	H13B—C13—H13C	109.5
C19—Mo2—C18	142.23 (19)	C15—C14—C18	107.1 (4)
C14—Mo2—C18	35.85 (16)	C15—C14—Mo2	72.3 (3)
C15—Mo2—C18	59.24 (17)	C18—C14—Mo2	73.7 (3)
C19—Mo2—C16	84.92 (19)	C15—C14—H14	126.2
C14—Mo2—C16	58.97 (17)	C18—C14—H14	126.2
C15—Mo2—C16	35.15 (16)	Mo2—C14—H14	126.2
C18—Mo2—C16	58.02 (17)	C16—C15—C14	107.8 (4)
C19—Mo2—C17	109.75 (19)	C16—C15—Mo2	74.4 (3)
C14—Mo2—C17	58.68 (17)	C14—C15—Mo2	71.6 (3)
C15—Mo2—C17	58.62 (16)	C16—C15—H15	125.9
C18—Mo2—C17	34.35 (17)	C14—C15—H15	125.9
C16—Mo2—C17	34.91 (17)	Mo2—C15—H15	125.9
C19—Mo2—S2^ii^	79.05 (14)	C15—C16—C17	108.1 (4)
C14—Mo2—S2^ii^	95.51 (13)	C15—C16—Mo2	70.4 (3)
C15—Mo2—S2^ii^	95.34 (12)	C17—C16—Mo2	72.9 (3)
C18—Mo2—S2^ii^	126.98 (12)	C15—C16—H16	125.9
C16—Mo2—S2^ii^	125.83 (13)	C17—C16—H16	125.9
C17—Mo2—S2^ii^	152.53 (11)	Mo2—C16—H16	125.9
C19—Mo2—S2	88.51 (14)	C18—C17—C16	108.4 (4)
C14—Mo2—S2	134.69 (13)	C18—C17—Mo2	71.7 (3)
C15—Mo2—S2	148.71 (12)	C16—C17—Mo2	72.2 (3)
C18—Mo2—S2	100.11 (12)	C18—C17—H17	125.7
C16—Mo2—S2	115.04 (12)	C16—C17—H17	125.7
C17—Mo2—S2	90.83 (12)	Mo2—C17—H17	125.7
S2^ii^—Mo2—S2	115.85 (3)	C17—C18—C14	108.5 (4)
C19—Mo2—Mo2^ii^	78.29 (15)	C17—C18—Mo2	74.0 (3)
C14—Mo2—Mo2^ii^	138.92 (12)	C14—C18—Mo2	70.5 (3)
C15—Mo2—Mo2^ii^	153.36 (12)	C17—C18—H18	125.6
C18—Mo2—Mo2^ii^	136.94 (13)	C14—C18—H18	125.6
C16—Mo2—Mo2^ii^	161.67 (12)	Mo2—C18—H18	125.6
C17—Mo2—Mo2^ii^	147.90 (12)	O2—C19—Mo2	175.0 (4)
S2^ii^—Mo2—Mo2^ii^	58.12 (3)	C21—C20—C25	119.4 (4)
S2—Mo2—Mo2^ii^	57.72 (3)	C21—C20—S2	122.3 (3)
C7—S1—Mo1	109.82 (14)	C25—C20—S2	118.0 (3)
C7—S1—Mo1^i^	117.34 (15)	C20—C21—C22	119.8 (4)
Mo1—S1—Mo1^i^	65.00 (3)	C20—C21—H21	120.1
C20—S2—Mo2^ii^	113.39 (14)	C22—C21—H21	120.1
C20—S2—Mo2	114.07 (14)	C23—C22—C21	121.9 (4)
Mo2^ii^—S2—Mo2	64.15 (3)	C23—C22—H22	119.0
C5—C1—C2	107.9 (4)	C21—C22—H22	119.0
C5—C1—Mo1	72.1 (3)	C22—C23—C24	118.2 (4)
C2—C1—Mo1	70.8 (3)	C22—C23—C26	121.8 (4)
C5—C1—H1	126.0	C24—C23—C26	120.0 (4)
C2—C1—H1	126.0	C25—C24—C23	120.3 (4)
Mo1—C1—H1	126.0	C25—C24—H24	119.8
C1—C2—C3	108.2 (4)	C23—C24—H24	119.8
C1—C2—Mo1	74.2 (3)	C24—C25—C20	120.3 (4)
C3—C2—Mo1	70.9 (3)	C24—C25—H25	119.8
C1—C2—H2	125.7	C20—C25—H25	119.8
C3—C2—H2	125.7	C23—C26—H26A	109.5
Mo1—C2—H2	125.7	C23—C26—H26B	109.5
C2—C3—C4	107.3 (4)	H26A—C26—H26B	109.5
C2—C3—Mo1	73.3 (3)	C23—C26—H26C	109.5
C4—C3—Mo1	72.5 (2)	H26A—C26—H26C	109.5
C2—C3—H3	126.1	H26B—C26—H26C	109.5
			
C6—Mo1—S1—C7	−33.3 (2)	Mo1^i^—Mo1—C5—C4	144.4 (2)
C3—Mo1—S1—C7	56.0 (2)	Mo1—S1—C7—C8	100.0 (4)
C4—Mo1—S1—C7	47.8 (2)	Mo1^i^—S1—C7—C8	28.6 (4)
C2—Mo1—S1—C7	90.4 (2)	Mo1—S1—C7—C12	−84.4 (3)
C5—Mo1—S1—C7	101.1 (3)	Mo1^i^—S1—C7—C12	−155.8 (3)
C1—Mo1—S1—C7	115.9 (2)	C12—C7—C8—C9	−0.8 (7)
S1^i^—Mo1—S1—C7	−111.75 (17)	S1—C7—C8—C9	174.7 (3)
Mo1^i^—Mo1—S1—C7	−111.75 (17)	C7—C8—C9—C10	0.8 (7)
C6—Mo1—S1—Mo1^i^	78.49 (13)	C8—C9—C10—C11	0.5 (7)
C3—Mo1—S1—Mo1^i^	167.74 (12)	C8—C9—C10—C13	−178.8 (4)
C4—Mo1—S1—Mo1^i^	159.57 (16)	C9—C10—C11—C12	−1.8 (7)
C2—Mo1—S1—Mo1^i^	−157.81 (13)	C13—C10—C11—C12	177.4 (4)
C5—Mo1—S1—Mo1^i^	−147.2 (2)	C8—C7—C12—C11	−0.5 (6)
C1—Mo1—S1—Mo1^i^	−132.37 (13)	S1—C7—C12—C11	−176.3 (3)
S1^i^—Mo1—S1—Mo1^i^	0.0	C10—C11—C12—C7	1.9 (7)
C19—Mo2—S2—C20	−28.4 (2)	C19—Mo2—C14—C15	11.2 (4)
C14—Mo2—S2—C20	125.5 (2)	C18—Mo2—C14—C15	−114.5 (4)
C15—Mo2—S2—C20	69.6 (3)	C16—Mo2—C14—C15	−37.4 (3)
C18—Mo2—S2—C20	114.6 (2)	C17—Mo2—C14—C15	−78.4 (3)
C16—Mo2—S2—C20	55.3 (2)	S2^ii^—Mo2—C14—C15	91.5 (3)
C17—Mo2—S2—C20	81.3 (2)	S2—Mo2—C14—C15	−133.1 (2)
S2^ii^—Mo2—S2—C20	−105.47 (16)	Mo2^ii^—Mo2—C14—C15	137.3 (2)
Mo2^ii^—Mo2—S2—C20	−105.47 (16)	C19—Mo2—C14—C18	125.8 (3)
C19—Mo2—S2—Mo2^ii^	77.08 (14)	C15—Mo2—C14—C18	114.5 (4)
C14—Mo2—S2—Mo2^ii^	−129.00 (17)	C16—Mo2—C14—C18	77.1 (3)
C15—Mo2—S2—Mo2^ii^	175.1 (2)	C17—Mo2—C14—C18	36.1 (3)
C18—Mo2—S2—Mo2^ii^	−139.93 (13)	S2^ii^—Mo2—C14—C18	−153.9 (3)
C16—Mo2—S2—Mo2^ii^	160.76 (14)	S2—Mo2—C14—C18	−18.6 (4)
C17—Mo2—S2—Mo2^ii^	−173.19 (13)	Mo2^ii^—Mo2—C14—C18	−108.1 (3)
S2^ii^—Mo2—S2—Mo2^ii^	0.0	C18—C14—C15—C16	0.2 (5)
C6—Mo1—C1—C5	−45.1 (4)	Mo2—C14—C15—C16	66.2 (3)
C3—Mo1—C1—C5	−79.2 (3)	C18—C14—C15—Mo2	−66.0 (3)
C4—Mo1—C1—C5	−36.8 (3)	C19—Mo2—C15—C16	73.2 (3)
C2—Mo1—C1—C5	−117.1 (4)	C14—Mo2—C15—C16	−115.3 (4)
S1—Mo1—C1—C5	−165.6 (2)	C18—Mo2—C15—C16	−77.0 (3)
S1^i^—Mo1—C1—C5	61.1 (3)	C17—Mo2—C15—C16	−36.7 (3)
Mo1^i^—Mo1—C1—C5	127.6 (2)	S2^ii^—Mo2—C15—C16	152.7 (3)
C6—Mo1—C1—C2	72.0 (4)	S2—Mo2—C15—C16	−22.9 (4)
C3—Mo1—C1—C2	37.8 (3)	Mo2^ii^—Mo2—C15—C16	147.8 (3)
C4—Mo1—C1—C2	80.2 (3)	C19—Mo2—C15—C14	−171.5 (3)
C5—Mo1—C1—C2	117.1 (4)	C18—Mo2—C15—C14	38.3 (3)
S1—Mo1—C1—C2	−48.5 (3)	C16—Mo2—C15—C14	115.3 (4)
S1^i^—Mo1—C1—C2	178.2 (2)	C17—Mo2—C15—C14	78.6 (3)
Mo1^i^—Mo1—C1—C2	−115.4 (3)	S2^ii^—Mo2—C15—C14	−92.0 (3)
C5—C1—C2—C3	−0.3 (5)	S2—Mo2—C15—C14	92.4 (3)
Mo1—C1—C2—C3	−63.2 (3)	Mo2^ii^—Mo2—C15—C14	−96.9 (4)
C5—C1—C2—Mo1	63.0 (3)	C14—C15—C16—C17	−0.6 (5)
C6—Mo1—C2—C1	−137.3 (3)	Mo2—C15—C16—C17	63.6 (3)
C3—Mo1—C2—C1	−116.2 (4)	C14—C15—C16—Mo2	−64.3 (3)
C4—Mo1—C2—C1	−77.8 (3)	C19—Mo2—C16—C15	−106.9 (3)
C5—Mo1—C2—C1	−36.5 (3)	C14—Mo2—C16—C15	38.4 (3)
S1—Mo1—C2—C1	136.6 (3)	C18—Mo2—C16—C15	80.7 (3)
S1^i^—Mo1—C2—C1	−3.1 (4)	C17—Mo2—C16—C15	117.0 (4)
Mo1^i^—Mo1—C2—C1	106.7 (3)	S2^ii^—Mo2—C16—C15	−34.3 (3)
C6—Mo1—C2—C3	−21.1 (3)	S2—Mo2—C16—C15	167.1 (3)
C4—Mo1—C2—C3	38.4 (3)	Mo2^ii^—Mo2—C16—C15	−130.5 (4)
C5—Mo1—C2—C3	79.7 (3)	C19—Mo2—C16—C17	136.1 (3)
C1—Mo1—C2—C3	116.2 (4)	C14—Mo2—C16—C17	−78.6 (3)
S1—Mo1—C2—C3	−107.2 (3)	C15—Mo2—C16—C17	−117.0 (4)
S1^i^—Mo1—C2—C3	113.1 (3)	C18—Mo2—C16—C17	−36.3 (3)
Mo1^i^—Mo1—C2—C3	−137.1 (2)	S2^ii^—Mo2—C16—C17	−151.4 (2)
C1—C2—C3—C4	0.4 (5)	S2—Mo2—C16—C17	50.1 (3)
Mo1—C2—C3—C4	−65.0 (3)	Mo2^ii^—Mo2—C16—C17	112.4 (4)
C1—C2—C3—Mo1	65.4 (3)	C15—C16—C17—C18	0.9 (5)
C6—Mo1—C3—C2	162.6 (3)	Mo2—C16—C17—C18	62.9 (3)
C4—Mo1—C3—C2	−114.9 (4)	C15—C16—C17—Mo2	−62.0 (3)
C5—Mo1—C3—C2	−77.6 (3)	C19—Mo2—C17—C18	−164.4 (3)
C1—Mo1—C3—C2	−36.9 (3)	C14—Mo2—C17—C18	−37.7 (3)
S1—Mo1—C3—C2	75.1 (3)	C15—Mo2—C17—C18	−80.3 (3)
S1^i^—Mo1—C3—C2	−124.0 (2)	C16—Mo2—C17—C18	−117.2 (4)
Mo1^i^—Mo1—C3—C2	99.1 (3)	S2^ii^—Mo2—C17—C18	−59.8 (4)
C6—Mo1—C3—C4	−82.6 (3)	S2—Mo2—C17—C18	106.9 (3)
C2—Mo1—C3—C4	114.9 (4)	Mo2^ii^—Mo2—C17—C18	96.0 (3)
C5—Mo1—C3—C4	37.3 (3)	C19—Mo2—C17—C16	−47.2 (3)
C1—Mo1—C3—C4	77.9 (3)	C14—Mo2—C17—C16	79.5 (3)
S1—Mo1—C3—C4	−170.0 (2)	C15—Mo2—C17—C16	36.9 (3)
S1^i^—Mo1—C3—C4	−9.1 (4)	C18—Mo2—C17—C16	117.2 (4)
Mo1^i^—Mo1—C3—C4	−146.0 (2)	S2^ii^—Mo2—C17—C16	57.4 (4)
C2—C3—C4—C5	−0.3 (5)	S2—Mo2—C17—C16	−136.0 (3)
Mo1—C3—C4—C5	−65.8 (3)	Mo2^ii^—Mo2—C17—C16	−146.8 (2)
C2—C3—C4—Mo1	65.5 (3)	C16—C17—C18—C14	−0.8 (5)
C6—Mo1—C4—C5	−148.6 (3)	Mo2—C17—C18—C14	62.4 (3)
C3—Mo1—C4—C5	115.6 (4)	C16—C17—C18—Mo2	−63.2 (3)
C2—Mo1—C4—C5	77.4 (3)	C15—C14—C18—C17	0.4 (5)
C1—Mo1—C4—C5	36.3 (3)	Mo2—C14—C18—C17	−64.7 (3)
S1—Mo1—C4—C5	129.4 (2)	C15—C14—C18—Mo2	65.1 (3)
S1^i^—Mo1—C4—C5	−70.1 (3)	C19—Mo2—C18—C17	24.4 (4)
Mo1^i^—Mo1—C4—C5	−111.1 (4)	C14—Mo2—C18—C17	116.9 (4)
C6—Mo1—C4—C3	95.8 (3)	C15—Mo2—C18—C17	78.3 (3)
C2—Mo1—C4—C3	−38.1 (3)	C16—Mo2—C18—C17	36.9 (3)
C5—Mo1—C4—C3	−115.6 (4)	S2^ii^—Mo2—C18—C17	150.1 (2)
C1—Mo1—C4—C3	−79.3 (3)	S2—Mo2—C18—C17	−76.4 (3)
S1—Mo1—C4—C3	13.8 (3)	Mo2^ii^—Mo2—C18—C17	−129.3 (3)
S1^i^—Mo1—C4—C3	174.4 (2)	C19—Mo2—C18—C14	−92.5 (4)
Mo1^i^—Mo1—C4—C3	133.3 (3)	C15—Mo2—C18—C14	−38.6 (3)
C2—C1—C5—C4	0.1 (5)	C16—Mo2—C18—C14	−80.0 (3)
Mo1—C1—C5—C4	62.2 (3)	C17—Mo2—C18—C14	−116.9 (4)
C2—C1—C5—Mo1	−62.1 (3)	S2^ii^—Mo2—C18—C14	33.2 (3)
C3—C4—C5—C1	0.2 (5)	S2—Mo2—C18—C14	166.7 (3)
Mo1—C4—C5—C1	−63.9 (3)	Mo2^ii^—Mo2—C18—C14	113.8 (3)
C3—C4—C5—Mo1	64.0 (3)	Mo2^ii^—S2—C20—C21	−104.6 (4)
C6—Mo1—C5—C1	152.3 (3)	Mo2—S2—C20—C21	−33.7 (4)
C3—Mo1—C5—C1	79.1 (3)	Mo2^ii^—S2—C20—C25	80.6 (3)
C4—Mo1—C5—C1	117.3 (4)	Mo2—S2—C20—C25	151.6 (3)
C2—Mo1—C5—C1	36.9 (3)	C25—C20—C21—C22	−2.0 (7)
S1—Mo1—C5—C1	24.3 (4)	S2—C20—C21—C22	−176.6 (3)
S1^i^—Mo1—C5—C1	−126.1 (3)	C20—C21—C22—C23	0.3 (7)
Mo1^i^—Mo1—C5—C1	−98.2 (3)	C21—C22—C23—C24	0.9 (7)
C6—Mo1—C5—C4	35.0 (3)	C21—C22—C23—C26	−178.2 (4)
C3—Mo1—C5—C4	−38.2 (3)	C22—C23—C24—C25	−0.5 (6)
C2—Mo1—C5—C4	−80.5 (3)	C26—C23—C24—C25	178.6 (4)
C1—Mo1—C5—C4	−117.3 (4)	C23—C24—C25—C20	−1.1 (7)
S1—Mo1—C5—C4	−93.0 (3)	C21—C20—C25—C24	2.3 (6)
S1^i^—Mo1—C5—C4	116.5 (3)	S2—C20—C25—C24	177.2 (3)

Symmetry codes: (i) −*x*+1, −*y*, −*z*+1; (ii) −*x*, −*y*+1, −*z*+1.
